# Post-transplant anti-thymocyte globulin exposure predicts graft-versus-host disease and donor chimerism outcomes in reduced intensity conditioning allogeneic stem cell transplantation

**DOI:** 10.3389/fimmu.2026.1879058

**Published:** 2026-07-22

**Authors:** Ray Mun Koo, Joanne Davis, Mandy Ludford-Menting, Eric Wong, Rachel Koldej, David Ritchie

**Affiliations:** 1 Faculty of Medicine, Dentistry and Health Sciences, The University of Melbourne, Melbourne, VIC, Australia; 2 Australian Cancer Research Foundation (ACRF) Translational Research Laboratory, The Royal Melbourne Hospital, Melbourne, VIC, Australia; 3 Clinical Haematology, Peter MacCallum Cancer Centre - Royal Melbourne Hospital, Melbourne, VIC, Australia; 4 Department of Clinical Haematology, Austin Health, Melbourne, VIC, Australia

**Keywords:** allogeneic stem cell transplantation (allo-SCT), anti-thymocyte globulin (ATG), chimerism after allo-HSCT, graft-versus-host disease (GVHD), haematological malignancies, pharmacodynamics (PD), pharmacokinetics, reduced intensity conditioning (RIC)

## Abstract

Although anti-thymocyte globulin (ATG) is commonly used as prophylaxis against graft-versus-host disease (GVHD) following allogeneic haematopoietic stem cell transplantation (alloSCT), there is considerable inter-patient variability in ATG exposure. The impact of ATG exposure in outcomes following reduced intensity conditioning (RIC)-alloSCT remains unknown. We explored the impact of post-transplant ATG exposure (PT-ATG-exp) in 68 adults with haematological malignancies undertaking first RIC-alloSCT with ATG (Thymoglobulin, total dose 4.5mg/kg, day -3 to -1 alloSCT). Post-transplant ATG exposure was determined by area under the curve (AUC) through analysis of sera collected at standardised timepoints. Post-transplant ATG exposure range was highly variable [46–420 arbitrary units (AU) per day/mL]. Day 0 ATG concentration and day 0–14 ATG AUC highly correlated with PT-ATG-exp (R = 0.93 and 0.99, respectively, *P* < 0.001). Using a Cox proportional hazards model, patients with PT-ATG-exp between 80–135 AU per day/mL had inferior 2-year GRFS compared to the remainder of the cohort [25% (95% CI, 11%-56%) vs 54% (95% CI, 40%-73%), *P* = 0.02; HR 2.01 (95% CI, 1.04-3.86, *P* = 0.04] due to higher incidence of grade III-IV acute GVHD [33% (95% CI, 16%-51%) vs 12% (95% CI, 4%-24%), *P* = 0.034; HR 2.95 (95% CI, 0.99-8.83, *P* = 0.05)]. Recipients with the highest PT-ATG-exp (quartile 4: 185–420 AU per ml/day) had the lowest incidence of grade III-IV acute GVHD (0% at 180-days) but also lowest median CD3^+^ chimerism (85%) and highest incidence of mixed donor chimerism (CD3^+^: 93% and CD3^-^: 33%) at day 100. In this analysis, PT-ATG-exp influenced post-transplant outcomes including GVHD incidence and donor chimerism following in RIC-alloSCT.

## Introduction

Allogeneic haematopoietic stem cell transplantation (alloSCT) is an effective treatment against haematological malignancies due to the immunologically-mediated graft-versus-tumour (GVT) effect (1, 2). However, the same alloreactive donor immune subsets that confer the GVT response can also precipitate graft-versus-host disease (GVHD) which is a major contributor of post-transplant morbidity and mortality (3, 4). Strategies that reduce GVHD while preserving the GVT effect are therefore crucial for the curative potential of alloSCT.

Rabbit anti-thymocyte globulin (ATG) is commonly used as prophylaxis against GVHD. By means of *in vivo* depletion of T cells from the stem cell graft, ATG reduces the incidence and severity of acute and chronic GVHD following alloSCT, translating into improvements in quality of life and reduction in GVHD-related mortality (5–10). Recipients of matched unrelated and haploidentical donors have derived significant benefit, with GVHD outcomes now comparable to those observed in matched related donor alloSCT (7, 11, 12).

However, a careful balance exists between preventing GVHD and maintain the GVT effect; excessive depletion of donor-derived T cells runs the risk of suppressing the beneficial GVT effect and increasing relapse. This balance is also context dependent, with key factors being transplant conditioning intensity and disease-related factors. For example, retrospective studies have suggested that reduced intensity conditioning (RIC)-alloSCT recipients receiving high-dose ATG may be at increased risk of disease relapse (13, 14), resulting in inferior survival in at least one cohort (14).

ATG dosing is most commonly weight-based and there is considerable inter-patient variability in ATG exposure (15, 16). Here, we report the findings of a retrospective pharmacokinetic-pharmacodynamic study of ATG in a cohort of patients with haematological malignancies undergoing alloSCT using a standardised RIC and ATG dosing regimen. We explored the key determinants of PT-ATG-exp in this patient population, and correlated PT-ATG-exp with key post-transplant and immunological engraftment outcomes.

## Patients and methods

Adult patients undergoing first RIC-alloSCT from a 10/10 HLA matched unrelated donor at the Royal Melbourne Hospital and Peter MacCallum Cancer Centre, Melbourne, Australia between August 2018 and February 2024 were included in this analysis. Conditioning was limited to fludarabine 125mg/m^2^ (25mg/m^2^/day, day -7 and -3) and melphalan 100-140mg/m^2^ (day -2). All patients received ATG, Thymoglobulin^®^ (Genzyme Corporation, Massachusetts, US) 4.5mg/kg administered between day -3 to day -1 (0.5mg/kg/day on day -3, and 2mg/kg/day on day -2 and -1), cyclosporin and short-course methotrexate. All patients received fungal, viral and pneumocystis prophylaxis, and patients who are high-risk for cytomegalovirus (CMV) reactivation (seropositive recipients) received high-dose valaciclovir prophylaxis. Monitoring of Epstein-Barr virus (EBV) and CMV DNA was by real-time polymerase chain reaction (PCR) twice weekly until day 100 alloSCT. Plasma samples for ATG concentration measurement (day 0, 14 and 30 of alloSCT) were prospectively collected after written consent was obtained. Flow cytometric analysis was utilised to determine ATG concentration at individual timepoints and expressed at arbitrary units per millilitre (AU/mL). The PT-ATG-exp (AU per day/mL) for each patient was determined by area under the curve (AUC), through summation of ATG exposure between day 0–14 and day 14-30. Description of flow cytometric analysis and ATG exposure calculation are detailed in **Supplementary Material**. Chimerism analysis was through PCR of short tandem repeat markers, on peripheral blood samples collected at day 30, 60 and 100 following alloSCT. The study was approved by the Human Research Ethics Committee of the Royal Melbourne Hospital (MH2023.187 and MH2018.017).

### Outcomes

The primary outcome was GVHD-free, relapse-free survival (GRFS), as this composite endpoint considers both impact of ATG on clinically significant GVHD which may lead to non-relapse mortality (NRM) and disease relapse due to possible deleterious effects of ATG on the GVT response. Secondary outcomes include OS, acute and chronic GVHD, disease relapse, NRM and donor-recipient chimerism. Overall survival (OS) was defined as survival status at time of last follow up. Non-relapse mortality was defined as death occurring prior to disease relapse or progression. Freedom from relapse, death, grade III-IV acute GVHD or moderate-severe chronic GVHD was used to define GRFS. Diagnosis and grading of acute and chronic GVHD were according to MAGIC (17) and National Institutes of Health consensus criteria (18) respectively. Mixed donor chimerism was defined as < 95% donor cells in T cell (CD3^+^) or myeloid (CD3^-^) fractions.

### Statistical analysis

Baseline characteristics were summarised using descriptive statistics. Spearman rank correlation was used to determine association between variables suspected to influence post-transplant ATG levels, with *P ≤* 0.05 considered statistically significant. The Kaplan-Meier method (19) was used to estimate the OS, GRFS and PFS, and cumulative incidence function by Fine and Gray (20) was used to estimate the incidence of GVHD, relapse and NRM. Landmark analyses were performed at the 2-year timepoint for all clinical outcomes, with the exception for acute GVHD (180 days). To identify the range of PT-ATG-exp which resulted in superior 2-year GRFS, a proportional hazard model was used, which plots the log relative hazard ratio of an event in relation to PT-ATG-exp. A Cox proportional hazards model was used to determine association between variables on GRFS, with *P ≤* 0.1 considered statistically significant – and multivariable analysis performed on all variables of statistical significance. Statistical analysis was performed using R analysis software (Comprehensive R Archive Network Project), SPSS Statistics 30 (SPSS Inc., Illinois, US) and GraphPad Prism for Apple (GraphPad Software, Massachusetts, US).

## Results

Sixty-eight patients were included with a median follow up of 668 days (range: 58-2181) (**Table 1**). Myeloid malignancies were the predominant transplant indication (75%), with the majority consisting of myelodysplasia (MDS, n=22). All patients with non-Hodgkin lymphoma (NHL, 15%) were in complete remission at time of alloSCT. The majority of patients (n=41, 60%) were CMV recipient seropositive. Median time to neutrophil and platelet engraftment was 23 days (range: 16-37) and 29 days (range: 15-125), respectively.

**Table 1:** Patient characteristics.

Characteristics	All (n=68)
Age, median years (range)	61 (20-71)
Gender, n (%)	
Male	41 (60)
Female	27 (40)
Disease, n (%)	
AML	20 (29)
MDS	22 (32)
MPN	9 (13)
NHL	15 (22)
CLL	1 (1)
HL	1 (1)
Disease risk index, n (%)	
Low	5 (7)
Intermediate	49 (72)
High	9 (13)
Not available	5 (7)
HCT-CI score, n (%)	
0	19 (28)
1-2	22 (32)
≥ 3	27 (40)
ECOG status, n (%)	
0	60 (88)
1	8 (12)
CD34^+^ dose (x10^6^/kg)	5.05 (1.7-8.04)
CD3^+^ dose (x10^6^/kg)	206 (17.5-639)
Lymphocyte count, (x10^9^/L), median (range)	
Prior to start of conditioning (day -7)	1 (0.2-6.4)
Prior to first dose of ATG (day -3)	0.2 (0-1.2)
Prior to graft infusion (day 0)	0 (0-0.1)

AML, Acute myeloid leukaemia; CLL, chronic lymphocytic leukaemia; ECOG, Eastern Co-operation Oncology Group; HCT-CI, haematopoietic cell transplantation comorbidity index; HL, Hodgkin lymphoma; MPN, myeloproliferative neoplasm.

For the entire cohort, the 2-year OS was 70% (95% CI, 60%-83%). Leading causes of death were GVHD (n=5, 25%) followed by sepsis (n=4, 20%) and progressive disease (n=3, 15%). Eight patients relapsed at a median of 179 days (range: 60-1089) resulting in a 2-year relapse incidence of 12% (95% CI, 5%-22%). Twenty-three patients (34%) developed acute GVHD at a median of 112 days (range: 40-180) with the majority (61%) with grade III-IV acute GVHD. Sixteen patients (24%) developed chronic GVHD at a median of 266 days (range: 160-403) and majority were moderate-severe chronic GVHD (63%). The 2-year GRFS for the entire cohort was 44% (95% CI, 33%-58%), and other key transplant outcomes are summarised in **Supplementary Table 1**. Thirty seven patients encountered CMV reactivation, resulting in a 6-month incidence of 54% (95% CI, 42%-65%) and almost all (97%) occurring in individuals who were CMV recipient seropositive. The 6-month incidence of EBV reactivation was 71% (95% CI, 58%-80%), and EBV reactivation requiring therapy was 34% (95% CI, 23%-45%).

### Post-transplant ATG exposure and clinical outcomes

A total of 204 plasma samples from all 68 patients were available for analysis. The median ATG concentrations on days 0, 14 and 30 post-alloSCT were 8.4 AU/mL (range: 1.4-20), 1.1 AU/mL (range: 0.28-4.1) and 0.32 AU/mL (range: 0.012-2.4), respectively and median PT-ATG-exp was 125 AU per day/mL (range: 47-420). Anti-thymocyte globulin exposure over day 0–14 and day 14-30, and according to quartiles are shown in **Supplementary Figure 1**. Variables with the strongest correlation to PT-ATG-exp were ATG exposure between day 0-14 (R = 0.993, *p* < 0.001) and ATG concentration on day 0 (R = 0.925, *p* < 0.001). Graft CD3^+^ content, recipient body weight and absolute lymphocyte count at time of first ATG administration did not correlate with PT-ATG-exp (**Supplementary Table 2**).

**Figure 1:**
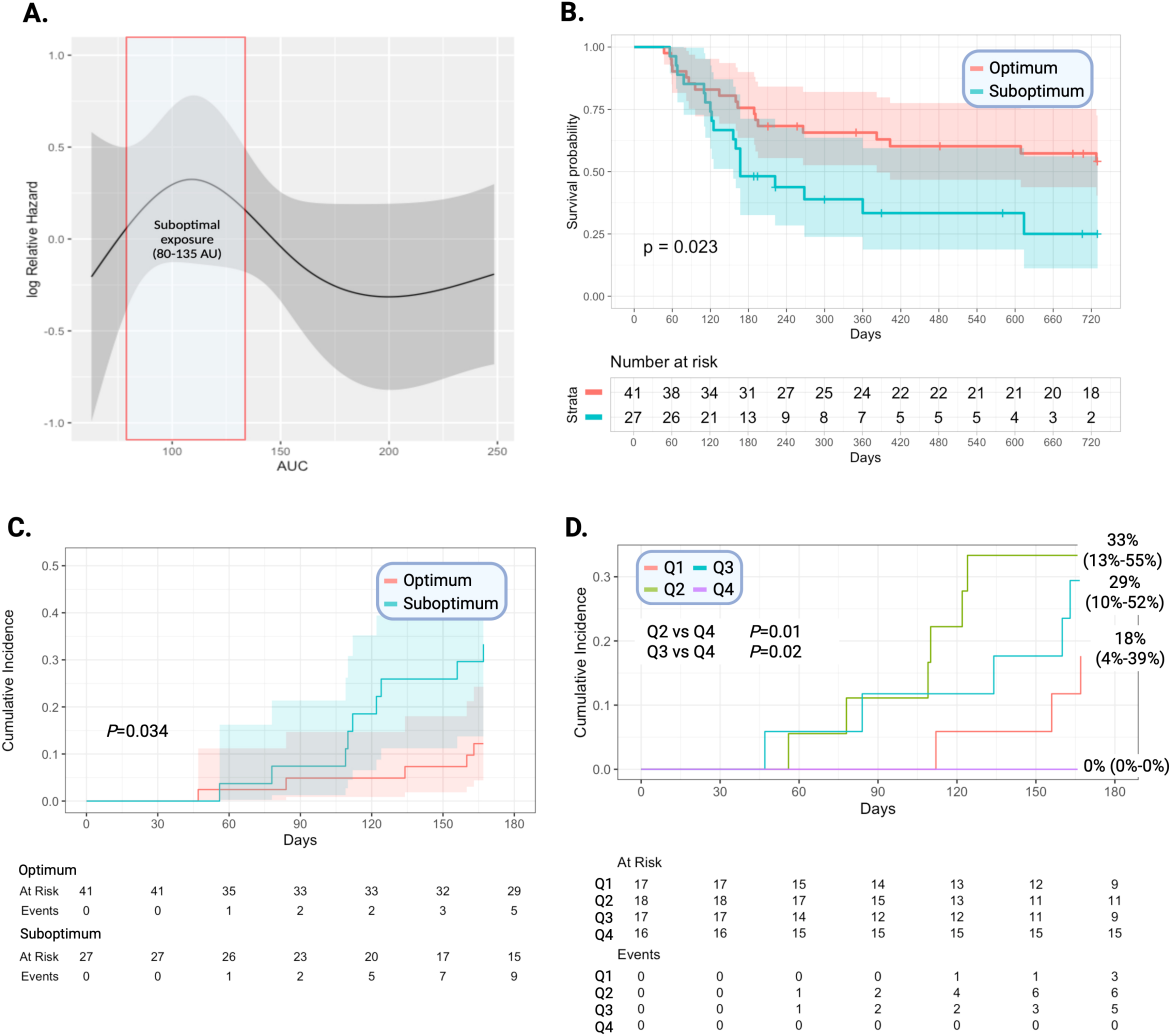
**(A)** GRFS risk according to PT-ATG-exp^*†*^. **(B)** Estimates of GRFS according to PT-ATG-exp. **(C)** Estimates of grade III-IV acute GVHD according to PT-ATG-exp. **(D)** Estimates of grade III-IV acute GVHD according to quartiles of PT-ATG-exp. Gray shaded areas represent 95% CI for log relative hazard. Red box represents ATG exposure range associated with inferior GRFS outcomes.

To determine the range of PT-ATG-exp associated with 2-year GRFS, the log relative hazard ratio for GRFS was plotted against PT-ATG-exp for the entire cohort (**Figure 1A**). The range of suboptimal PT-ATG-exp was identified at 80–135 AU per day/mL, whereby individuals within this ATG exposure range had inferior 2-year GRFS [25% (95% CI, 11%-56%) vs 54% (95% CI, 40%-73%), *P* = 0.02 and HR 2.01 (95% CI, 1.04-3.86, *P* = 0.04] (**Figure 1B**) due to a higher incidence of grade III-IV acute GVHD [33% (95% CI, 16%-51%) vs 12% (95% CI, 4%-24%), *P* = 0.034 and HR 2.95 (95% CI, 0.99-8.83, *P* = 0.05] (**Figure 1C**). However, individuals within this suboptimal PT-ATG-exp range did not have inferior chronic GVHD, disease relapse or survival outcomes (**Supplementary Table 3**).

Associations between quartiles (Q1-4) of PT-ATG-exp [Q1 (46–94 AU per day/mL), Q2 (95–124 AU per day/mL), Q3 (125–184 AU per day/mL) and Q4 (185–420 AU per day/mL)] (**Supplementary Figure 1C**) with transplant outcomes were then examined. Whilst there was no difference in estimated 2-year GRFS when PT-ATG-exp was stratified according to quartiles (**Supplementary Table 3**), the incidence grade III-IV acute GVHD was significantly lower in Q4 compared to Q2 [33% (95% CI, 13%-55%), *P* = 0.01) and Q3 [29% (95% CI, 10%-52%), *P* = 0.02] (**Figure 1D**). When stratified according to quartiles, there was no significant association between PT-ATG-exp and disease relapse, OS and NRM (data not shown). For chronic GVHD, 2-year incidence was greater in Q4 compared to Q3 [44% (95% CI, 16%-69%) vs 12% (95% CI, 2%-32%), *P* = 0.05] but not moderate-severe chronic GVHD [12% (95% CI, 2%-32%) vs 23% (95% CI, 5%-48%), *P* = 0.5]. Although there was no difference in CMV or EBV reactivation, for individuals with high-risk of CMV reactivation (recipient seropositive and donor seronegative, or donor and recipient seropositive), reactivation was greater in Q4 compared to Q3 [100% (95% CI, 100%-100%) vs 87% (95% CI, 15%-99%), *P* = 0.05].

### Post-transplant ATG exposure and chimerism outcomes

**Figure 2:**
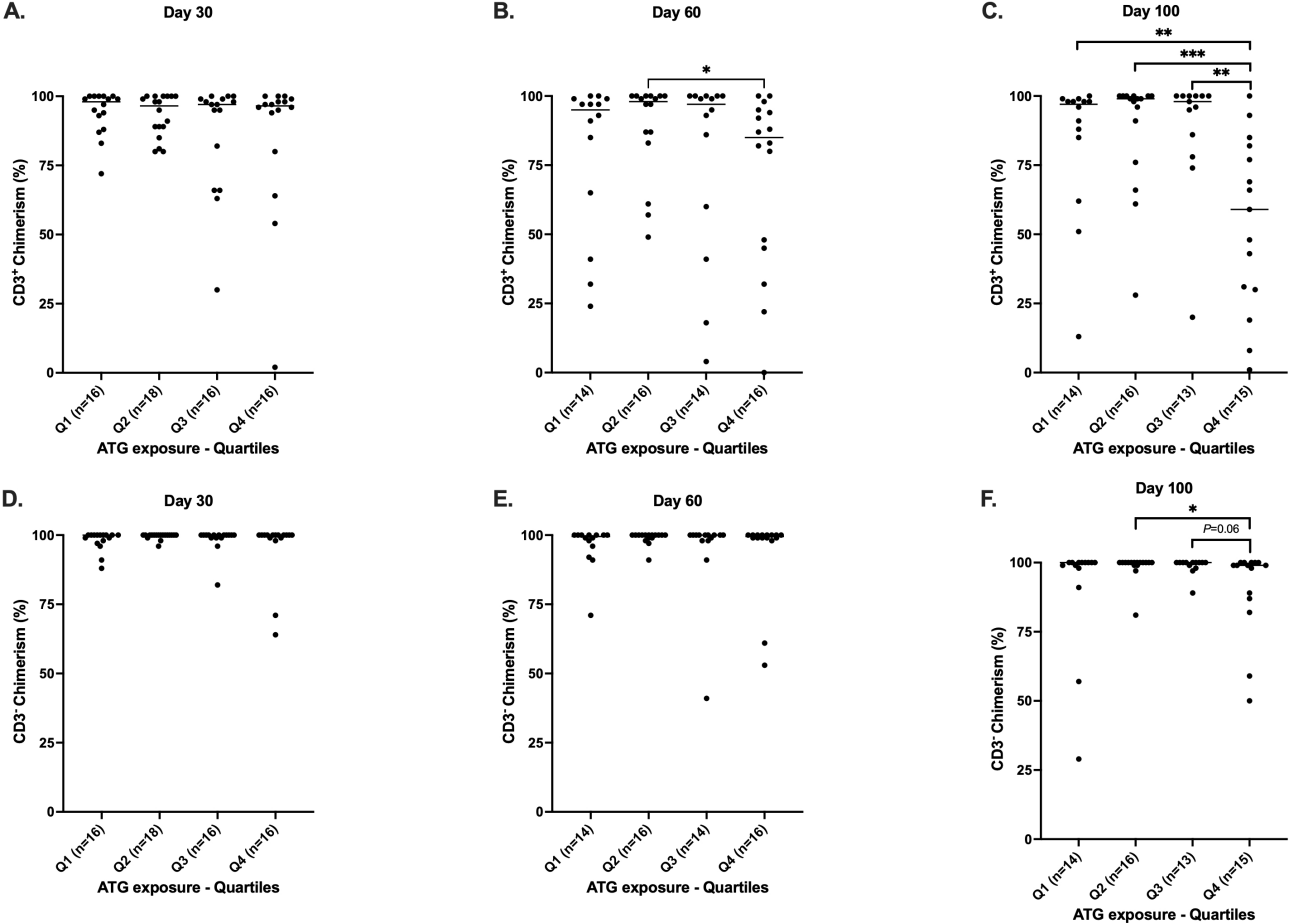
Donor chimerism according to quartiles. **(A)** CD3^+^ chimerism at day 30. **(B)** CD3^+^ chimerism at day 60. **(C)** CD3^+^ chimerism at day 100. **(D)** CD3^-^ chimerism at day 30. **(E)** CD3^-^ chimerism at day 60. **(F)** CD3^-^ chimerism at day 100. Mann-Whitney test. *P<0.05, **P<0.005, ***P<0.001.

Donor chimerism results were available for 66 patients (97%) at day 30, 60 patients (88%) at day 60 (88%) and 58 patients (85%) at day 100, with a median CD3^+^ donor chimerism of 97% (range: 2-100), 94% (range: 0-100) and 94% (range: 1-100%), respectively. Whilst median CD3^+^ donor chimerism were similar at day 30 (**Figure 2A**), at day 60 median CD3^+^ donor chimerism in Q4 was significantly lower compared to Q2 [85% (range: 0-100) vs 98% (range: 49-100), *P* = 0.03] (**Figure 2B**). At day 100, median CD3^+^ donor chimerism for Q4 [85% (range: 0-100)] was significantly lower compared to CD3^+^ donor chimerism in Q1 [97% (range: 13-100), *P* = 0.005], Q2 [99% (range: 28-100), *P* = 0.001] and Q3 [98% (range: 20-100), *P* = 0.003] (**Figure 2C**). In addition, PT-ATG-exp was significantly associated with day 100 CD3^+^ MDC (*P* < 0.001), with day 100 CD3^+^ MDC most frequently seen in Q4 (n=14, 93%) compared to Q1 (n=6, 43%), Q2 (n=5, 31%) and Q3 (n=4, 31%). For CD3^-^ donor chimerism, median chimerism at day 30, 60 and 100 were similar (100%). Whilst median CD3^-^ donor chimerism were similar at day 30 and 60 (**Figures 2D, E**), at day 100, median CD3^-^ donor chimerism in Q4 was significantly lower compared to Q2 (**Figure 2F**).

## Discussion

In this retrospective analysis, we examined the impact of PT-ATG-exp on RIC-alloSCT outcomes in a cohort of homogenously treated patients. Despite utilising a standardised weight-based ATG dosing strategy, we demonstrate significant heterogeneity in PT-ATG-exp. We identified a range of PT-ATG-exp associated with inferior GRFS due to more frequent grade III-IV acute GVHD. We also showed that individuals with the greatest PT-ATG-exp are protected against grade III-IV acute GVHD but at the expense of poorer donor immunological engraftment, characterised by high incidence of CD3^+^ MDC and lower median CD3^+^ donor chimerism.

We found that the central determinant of PT-ATG-exp was the concentration of active ATG on day of graft infusion, while biological factors such as CD3^+^ cell dose and recipient body weight correlated poorly with PT-ATG-exp. In a study of myeloablative alloSCT recipients, Jamani et al. highlighted the importance of recipient body weight as a determinant of PT-ATG-exp – a finding not identified by Admiraal et al. (15, 16). It is important to note that these studies, and ours, utilised different ATG dosing strategies, ATG doses and conditioning agents – factors which affect the availability of circulating active ATG following graft infusion. Whilst both Admiraal et al. and Jamani et al. identified that the absolute lymphocyte count prior to first ATG dose influences PT-ATG-exp (15, 16), our study did not. We note that our patients were markedly lymphopenic (median: 0.2x10^9^/L) at time of first ATG infusion due to high dose fludarabine pre-treatment, therefore making any correlative analysis impossible. In fact, we would argue that the absolute lymphocyte count is a poor surrogate for the target of active ATG. As purified polyclonal antibodies directed against human thymic tissue, ATG has high specificity not only against naive T cells, but also against various non-T cell targets such as macrophages, monocytes, B cells and stromal tissue (21, 22).

Our study showed that recipients with a suboptimum PT-ATG-exp range of 80–135 AU per day/mL had inferior GRFS due to an increased incidence of grade III-IV acute GVHD without impacting survival or disease relapse. The optimal range of ATG exposure in our study differs from thar reported by Admiraal et al. highlighting that the identification of the optimal ATG exposure is context dependent (15). Our study therefore demonstrates the importance of developing pharmacokinetic-pharmacodynamic studies which are population-specific, considering factors such as the schedule of ATG used, conditioning intensity, GVHD prophylaxis and disease related factors.

As with previous authors (16), we demonstrated that recipients with the highest quartile of PT-ATG-exp had superior grade III-IV acute GVHD outcomes. However, this occurred at the expense of impaired donor immune reconstitution, as signified by lower CD3^+^ donor chimerism at day 60 and day 100, and a greater incidence of CD3^+^ MDC at day 100. Surprisingly, the incidence of chronic GVHD in this cohort [44%, (95% CI, 16%-69%)] was the highest compared to other quartiles. A potential explanation is that immunomodulatory attempts to improve CD3^+^ donor chimerism may have led to the development of chronic GVHD in these individuals. Whilst cyclosporin doses and timing of immunosuppression withdrawal was not collated for this analysis, we note that 67% among all patients who received donor lymphocyte infusion (DLI) for CD3^+^ MDC in this study were from the Q4 cohort and 75% of these individuals subsequently developed GVHD, thus potentially supporting our hypothesis.

Prior authors have correlated patterns of viral reactivation with PT-ATG-exp (23). In a study of myeloablative haploidentical alloSCT, Wang et al. demonstrated that high PT-ATG-exp (defined as above the median) was associated with an increased incidence of CMV reactivation but not EBV reactivation. We demonstrate similar findings, although higher CMV reactivation incidences was only noted in individuals who are recipient CMV seropositive, despite appropriate CMV prophylaxis. It is important to note that Wang et al. did not include data on CMV seropositivity or use of prophylaxis in their patients, hence it may be plausible that high PT-ATG-exp is a risk factor for CMV reactivation only in CMV seropositive patients.

Our analysis has several limitations. The relatively small patient numbers limit the ability to further subclassify recipients into those who had superior GRFS outcomes despite low PT-ATG-exp (n=10). Whilst our study did not indicate any impact of PT-ATG-exp on survival, chronic GVHD and disease relapse, a larger patient sample size may provide additional insights. Despite only including patients treated with a common RIC and ATG prophylaxis dosing regimen, this analysis incorporated patients with various haematological malignancies, including more indolent diseases. This may account for the low incidence of disease relapse in this study, thus limiting our ability to examine disease relapse robustly as an outcome of interest.

Our study highlights the growing importance of PT-ATG-exp as an important biomarker of alloSCT outcomes, particularly the development of clinically significant acute GVHD and CD3^+^ MDC. We show that ATG concentration measured on day 0 is the strongest predictor of PT-ATG-exp and may therefore represent an important and practical timepoint for ATG measurement in routine clinical practice. While therapeutic drug monitoring of ATG has been shown to be possible within the scope of clinical trials (24, 25), implementing this within the workflow of routine clinical practice may prove challenging due to the labour-intensive nature of this bioassay. Instead, our study suggests that a single timepoint for ATG measurement (day 0) may obviate the need for a more complete determination of exposure, which would thus allow interventions to be undertaken in a timely fashion. For patients with anticipated poorer acute GVHD outcomes due to low ATG exposure, the addition of low dose post-transplant cyclophosphamide (PTCy) on day +3 and +4 may prevent clinically significant GVHD from occurring. Using additional doses of ATG following graft infusion must be cautioned, as we have demonstrated high incidence of CD3^+^ MDC in patients with very high ATG PT-ATG-exp. For patients anticipated to have the highest PT-ATG-exp, early withdrawal of immunosuppression to reverse CD3^+^ MDC could be considered, however further validation in a larger patient cohort is required.

Whilst PTCy has now emerged as the agent of choice for GVHD prophylaxis in matched donor transplantation (26, 27), we highlight that ATG remains the gold standard for alloSCT in severe aplastic anaemia (28), forms the backbone of haploidentical alloSCT in China (29) and is widely utilised in solid organ transplantation for both the prevention and treatment of graft rejection (30). As the field of transplantation continues to embrace the concept of personalized medicine, we encourage other investigators to explore the impact of ATG pharmacokinetics-pharmacodynamics on clinical and immunological outcomes in these respective settings.

## Data Availability

The raw data supporting the conclusions of this article will be made available by the authors, without undue reservation.
