# Supplementary material for: Post-transplant anti-thymocyte globulin exposure predicts graft-versus-host disease and donor chimerism outcomes in reduced intensity conditioning allogeneic stem cell transplantation
Source: Front Immunol. 2026 Jul 22;17:1879058. doi: 10.3389/fimmu.2026.1879058 (PMC13437416; doi:10.3389/fimmu.2026.1879058)
Supplement: Supplementary file 1 [file SupplementaryFile1.docx]

**SUPPLEMENTARY MATERIAL**

**MEASUREMENT OF ATG CONCENTRATION**

**Sample Preparation and Analysis**

Peripheral blood samples of patients were collected in EDTA and processed within 3 hours of collection to obtain patient plasma. Serial dilutions of ATG (Sanofi Genyzme; Massachusetts, US) in a range of 20 to 0.0303AU/mL were used for a calibration curve. All samples were stored at -80 degrees Celsius until time of analysis.

Eighty-five microliters of phosphate buffered saline containing Jurkat T cells (ATCC clone E6-1, In Vitro Technologies, Victoria, Melbourne, Australia) were added to a 96-well microplate [approximately 10x10^6^ cells/well], followed by fifteen microliter of corresponding calibration standard or patient plasma. Following 2 hours of incubation at room temperature, cells were washed and centrifuged twice, and the resulting cell pellet was incubated with PE donkey-anti-rabbit IgG antibody (BioLegend; California, US) at 4 degrees Celsius for 20 minutes in the dark. Further wash and centrifugation steps were performed, and cell pellets were fixed prior to flow cytometric acquisition on a LSRFortessa™ 5 laser flow cytometer (BD Biosciences; New Jersey, US). The excitation wavelength used during sample testing as 582nm and the emission wavelength was between 560-580nm. Detection speed was 1000 cells/second. The fluorescence was set to the geometric mean fluorescence intensity (MFI) of the PE area. Analysis was performed using FlowJo (BD Biosciences; New Jersey, US). A standard curve of the MFI of the PE area versus concentration of active ATG was generated using an asymmetrical nonlinear regression model in the form of a five-parameter logistic equation (5PL) on PRISM (GraphPad; Massachusetts, US). Corresponding samples were interpolated from standard curve with a 95% confidence interval.

**SUPPLEMENTARY RESULTS**

**Supplementary Table 1.** Post-transplant outcomes of the entire cohort

| **Outcomes, % (95% CI)** | **All (n=68)** |
| --- | --- |
| OS, 2-year | 70 (60-83) |
| PFS, 2-year | 65 (54-78) |
| NRM, 2-year | 23 (13-35) |
| Relapse, 2-year | 12 (5-21) |
| RRM, 2-year | 6 (2-14) |
| Acute GVHD (all), 180-day | 34 (23-45) |
| Grade II-IV acute GVHD, 180-day | 26 (17-37) |
| Grade III-IV acute GVHD, 180-day | 23 (14-35) |
| Chronic GVHD (all), 2-year | 26 (16-38) |
| Moderate to severe chronic GVHD, 2-year | 17 (8-27) |
| GRFS, 2-year | 44 (33-58) |

**Supplementary Figure 1.** **(A)** ATG concentration at day 0, 14 and 30 post-transplant. **(B)** Post-transplant ATG exposure for the entire cohort. **(C)** Post-transplant ATG exposure stratified to quartiles.

**Supplementary Table 2.** Variables impacting post-transplant ATG exposure

|  | **Correlation Coefficient, R** | ***P^ⴕ^*** |
| --- | --- | --- |
| Day 0 ATG concentration, AU/mL | 0.925 | **<0.001** |
| Day 14 ATG concentration, AU/mL | 0.877 | **<0.001** |
| Day 30 ATG concentration, AU/mL | 0.764 | **<0.001** |
| Day 0-14 ATG exposure, AU x days/mL | 0.993 | **<0.001** |
| Day 14-30 ATG exposure, AU x days/mL | 0.873 | **<0.001** |
| CD34^+^ cell dose, x10^6^/kg | -0.111 | 0.4 |
| CD34^+^ cell dose (total), x10^6^ | -0.205 | 0.09 |
| CD3^+^ cell dose, x10^6^/kg | 0.012 | 0.9 |
| CD3^+^ cell dose (total), x10^6^ | -0.028 | 0.8 |
| Weight, kg | 0.003 | 1.0 |
| Height, m | -0.085 | 0.5 |
| BMI, kg/m^2^ | 0.074 | 0.5 |
| BSA, m^2^ | -0.051 | 0.7 |
| Day -7 ALC, x10^9^/L | -0.119 | 0.3 |
| Day -3 ALC, x10^9^/L | -0.1 | 0.4 |
| Day 0 ALC, x10^9^/L | -0.2 | 0.1 |

^ⴕ^Spearman rank correlation test

**Supplementary Table 3.** Univariate analysis of post-transplant outcomes of the cohort stratified to ATG exposure

| **Outcomes, % (95% CI)** | **Suboptimal exposure range (n=27)** | **Optimal exposure range (n=41)** | **Hazard ratio**  **(95% CI)** | ***P*** |
| --- | --- | --- | --- | --- |
| GRFS, 2-year | 25 (11-56) | 54 (40-73) | 2.14 (1.10-4.17) | **0.02** |
| Acute GVHD (all), 180 days | 44 (25-62) | 27 (14-44) | 1.8 (0.78-4.01) | 0.2 |
| Grade II-IV acute GVHD, 180 days | 37 (19-55) | 20 (9-33) | 1.98 (0.78-5.04) | 0.1 |
| Grade III-IV acute GVHD, 180 days | 33 (16-51) | 12 (4-24) | 2.95 (0.99-8.83) | **0.05** |
| OS, 2-year | 65 (48-87) | 74 (62-90) | 1.65 (0.67-4.07) | 0.3 |
| NRM, 2-year | 32 (14-50) | 18 (8-32) | 2.08 (0.75-5.77) | 0.3 |
| Relapse, 2-year | 14 (3-33) | 11 (3-24) | 1.44 (0.32-6.56) | 0.6 |
| RRM, 2-year | 4 (0-16) | 8 (2-19) | 0.69 (0.07-6.63) | 0.7 |
| PFS, 2-year | 54 (37-80) | 71 (58-88) | 1.83 (0.79-4.24) | 0.2 |
| Chronic GVHD, 2-year | 30 (12-49) | 24 (11-38) | 1.81 (0.67-4.88) | 0.2 |
| Moderate to severe chronic GVHD, 2-year | 22 (7-41) | 13 (5-26) | 2.35 (0.67-8.20) | 0.2 |

**REFERENCES**

1.	Liu X, Huang C, Cao X, Yang X, Li S, Jiang S, et al. A fully validated flow cytometry method to quantitatively analyze active rATG in human serum and its application in pharmacokinetic study for therapeutic drug monitoring. J Pharm Biomed Anal. 2023;234:115483.
